# Sex perspective on mandatory admission in acute psychotic patients

**DOI:** 10.1192/j.eurpsy.2024.1694

**Published:** 2024-08-27

**Authors:** M. Delgado-Marí, À. Fernández-Ribas, A. Toll Privat, M. Giralt-López, B. Jiménez-Fernández, N. V. Motta-Rojas, J. Cuevas-Esteban

**Affiliations:** Psychiatry, Hospital Universitari Germans Trias i Pujol, Badalona, Spain

## Abstract

**Introduction:**

Psychotic disorders are strongly linked to a higher risk of mandatory hospitalization, often affecting men more, though some studies report the opposite. Recent investigations also show a higher rate of involuntary admissions in younger individuals. Knowledge in this area is still limited despite extensive research.

**Objectives:**

Analyze whether there is an association between sex and age with involuntary admissions of individuals with psychotic disorders.

**Methods:**

Retrospectively, 254 people with psychotic disorders admitted between 2018-2023 to the adult psychiatric inpatient unit at Hospital Universitari Germans Trias i Pujol were selected, collecting their nature of admission, sex, age, and discharge diagnosis. Comparisons between voluntary and involuntary admissions, with respect to sex and age variables, were conducted using independent sample t-tests, Mann-Whitney U tests, Fisher’s exact test, and chi-square tests. A logistic regression model was used to identify variables significantly associated with mandatory admission.

**Results:**

In both the male and female groups, there were no statistically significant differences in terms of the mean age at admission (p = 0.162) or the nature of admission (p = 0.586) (** Table 1**). When analyzing the voluntary nature of admission based on age and sex, statistically significant differences were only found in the female group (p = 0.01), resulting in a 9.18 year age difference among those admitted voluntarily (** Table 2**). The model that best predicted the probability of involuntary admission in individuals with psychotic disorders included the sex variable (OR = 4.88) and the interaction between sex and age (OR = 0.97) (** Table 3**).

**Table 1:** Differences between sex regarding voluntariness of patients with psychotic disorders.

	Male	Female	*p value*
**N (%)**	122 (48%)	132 (52%)	
**Age, m (SD)**	38.39 (16.64)	44.15 (18.44)	0.162
**Admissions, N (%)**			
Voluntary	38 (31.1%)	37 (28.0%)	0.586
Involuntary	84 (68.9%)	95 (72.0%)

**Table 2:** Analysis of voluntariness by sex and age.

**Age, m (SD)**	Voluntary	Involuntary	***p value***
Male	37.45 (16.38)	38.81 (16.84)	0.677
Female	50.76 (18.19)	41.58 (17.98)	0.01*
Total	44.01 (18.44)	40.28 (17.46)	0.127

**Table 3:** Predictors of involuntariness in psychotic patients: Logistic regression model (ENTER METHOD).

Predictor	-2log likelihood	Nagelkerke R2	x2 (df*)	OR* (95% CI*)	*p value*
	301.22	0.039	0.03 (1)		
Age				1.01 (0.98; 1.03)	0.674
Sex				4.88 (1.15; 20.72)	0.032*
Age x Sex Interaction				0.97 (0.94; 0.99)	0.046*

**Conclusions:**

Young women with psychotic disorders face a higher risk of involuntary admissions, emphasizing the need for gender-specific strategies to improve care of these patients.

**Disclosure of Interest:**

None Declared

